# Mechanistic Insights into Selenium-Induced Tolerance of Cucumber (*Cucumis sativus* L.) Seedlings to Alkaline Stress

**DOI:** 10.3390/plants15152271

**Published:** 2026-07-24

**Authors:** Wenjing Nie, Xiangyu Wang, Peng Qiao, Haiyang Zhang, Junlin Li, Rao Fu, Haiman Ge, Weijun Yin, Chi Zhang

**Affiliations:** 1 Yantai Engineering Research Center for Plant Stem Cell Targeted Breeding, Shandong Engineering Research Center of Functional Crop Germplasm Innovation and Cultivation Utilization, Shandong Institute of Sericulture, Yantai 265503, Chinagehainan@163.com (H.G.); 2 National Center of Technology Innovation for Comprehensive Utilization of Saline-Alkali Land, Dongying 265503, China

**Keywords:** cucumber, alleviating effect, selenium, alkaline stress

## Abstract

Saline–alkali stress severely restricts cucumber (*Cucumis sativus* L.) growth by disrupting ion balance, water status, photosynthesis, and redox homeostasis. Here, we examined the effects of exogenous selenium (Se) on cucumber seedlings exposed to NaHCO_3_ stress. Se supplementation improved plant growth and root activity and partly restored photosynthetic performance by maintaining chlorophyll content, gas exchange, and chlorophyll fluorescence. Se reduced oxidative injury through lower ROS and MDA levels and by enhancing antioxidant enzyme activities together with the AsA–GSH cycle. In parallel, Se moderated ion toxicity by limiting Na^+^ accumulation, increasing K^+^, Ca^2+^, and Mg^2+^ uptake, and stimulating H^+^-ATPase and H^+^-PPase activities. Enhanced TCA cycle activity and organic acid accumulation suggested improved energy metabolism and ionic regulation. Se also promoted osmotic adjustment via soluble sugars and proline, and upregulated aquaporin genes (*PIP1;2* and *PIP2;4*) to sustain water transport. Moreover, Se increased salicylic acid levels by upregulating *CsPAL* and *CsICS*, pointing to a role of SA signaling in Se-induced tolerance.

## 1. Introduction

Saline–alkaline stress (encompassing both salt and alkaline stress) is a major abiotic factor limiting sustainable agricultural development worldwide. Excessive salt and elevated pH, acting individually or in combination, trigger osmotic stress, oxidative damage, ion toxicity, and nutrient imbalances, thereby inhibiting normal plant growth and metabolism and ultimately reducing crop yield and quality [1,2]. In natural environments, salt accumulation and soil alkalization often occur simultaneously, and their combined impact is generally more severe than either stress alone, leading to pronounced disruption of plant physiological processes and ecosystem stability [2]. Globally, it is estimated that about 7% of the total land area and 33% of arable land are threatened by salinization [3], with the affected area continuing to expand. In China, salinealkaline soils exceed 100 million hectares and are increasing annually by approximately 10% [4]. This trend not only poses a serious threat to staple and cash crop production but also represents a major obstacle to the advancement of protected cultivation and modern farming systems.

Research on plant responses to saline–alkaline conditions has concentrated primarily on salt stress, while combined salt–alkali stress has received comparatively little attention. Yet under field conditions, salt and alkali stresses often coexist, and their joint effects on photosynthesis, ion regulation, antioxidant defense, and metabolic pathways are more complex and deleterious [2,5]. High Na^+^ levels directly disturb cellular ion homeostasis and compete with K^+^ uptake, thereby impairing metabolic balance [4]. Meanwhile, elevated pH decreases the availability of essential nutrients such as Ca^2+^, Mg^2+^, Fe, and P, further aggravating nutrient deficiencies [5,6,7]. Consequently, elucidating plant tolerance mechanisms under combined salt–alkali stress, identifying key genes and regulatory networks, and developing strategies to enhance tolerance are of considerable theoretical and practical significance. Such advances are crucial for promoting sustainable agriculture, ensuring food security, and improving the utilization of saline–alkaline lands [8,9,10].

Selenium (Se) is a trace micronutrient essential for humans and animals but generally not required for higher plants [11]. However, a wide range of studies have shown that suitable Se supplementation can improve plant growth, development, and resilience to diverse abiotic challenges [12]. The main inorganic forms of Se available to plants are selenate (SeO_4_^2−^) and selenite (SeO_3_^2−^). While selenate is readily soluble and taken up by roots, selenite is more strongly adsorbed by soil particles, which often restricts its bioavailability [12]. When supplied at appropriate concentrations, Se has been reported to stimulate biomass accumulation, enhance photosynthetic efficiency, and mitigate the negative effects of environmental stresses such as salinity, drought, heat, and heavy metal toxicity by strengthening antioxidant defenses [13,14,15,16,17,18,19]. These protective effects are associated with enhanced antioxidant defense, improved selenium uptake and transport, and the regulation of stress-related metabolic processes (e.g., superoxide dismutase, peroxidase, and catalase), reducing membrane lipid peroxidation, and restricting the overaccumulation of reactive oxygen species (ROS) such as H_2_O_2_ and superoxide anion (O_2_∙^−^), as indicated by lower malondialdehyde (MDA) accumulation, thereby preserving membrane integrity and cellular stability [20,21,22]. Furthermore, Se supplementation has been shown to improve photosynthetic performance, ion homeostasis, and stress adaptation under saline conditions, indirectly supporting plant adaptation and productivity. On the other hand, excessive Se can cause phytotoxicity in many species [23,24,25,26]. Nevertheless, when applied within an appropriate concentration range, Se effectively enhances plant stress tolerance, highlighting its potential importance in agricultural stress management [27,28].

Plants absorb Se from the environment through both roots and leaves. Under natural conditions, roots mainly take up selenate, selenite, and small amounts of organic selenium compounds [29]. Because selenate is structurally similar to sulfate, its uptake is mediated by high-affinity sulfate transporters [30,31,32,33]. In contrast, the mechanism of selenite absorption is more complex and may involve anion channels or cotransport systems [30,31,32,33]. Foliar application of Se fertilizers has also been confirmed as an effective supplementation strategy. For instance, Wang et al. (2020) [34] reported that foliar spraying with sodium selenate or different selenium forms significantly increased Se accumulation in maize, with sodium selenate showing greater efficacy. Similarly, Min et al. (2020) [35] showed that selenium accumulation in wheat varied with selenium form, application stage, and dosage. In cucumber seedlings, appropriate sodium selenite application has been shown to improve cadmium tolerance by enhancing antioxidant enzyme activity and reducing H_2_O_2_ and MDA levels [36,37]. Selenium is an essential constituent of glutathione peroxidase (GSH-Px) [38]. Although numerous studies have confirmed the role of Se in mitigating salt stress [39,40], its mechanism of action under alkaline stress or combined salt–alkali stress remains largely unclear.

Cucumber (*Cucumis sativus* L.), a salt-sensitive crop with the largest cultivated area in protected agriculture worldwide [41], is highly susceptible to saline–alkali conditions, which impair ion balance, photosynthesis, and root function, leading to reduced yield and quality [42]. Therefore, elucidating its physiological and metabolic responses under stress has both theoretical and practical significance. In this study, cucumber seedlings under NaHCO_3_ stress were used to investigate the alleviating effects of exogenous Se across multiple levels, including growth, photosynthetic performance, antioxidant defense, ion homeostasis, energy metabolism, osmotic regulation, and SA pathway-related gene expression. A hydroponic system with short-term stress exposure was adopted to minimize environmental variability and facilitate the analysis of early physiological and molecular responses to Se under alkaline stress. The results provide insights into the mechanisms by which Se enhances salt–alkali stress tolerance and offer a theoretical basis and practical reference for the cultivation of salt-tolerant cucumber varieties and the efficient production of vegetables in saline–alkaline areas.

## 2. Results

### 2.1. Exogenous Se Alleviates Growth Inhibition and Photosynthetic Suppression Under Alkaline (NaHCO_3_) Stress

**Figure 1:**
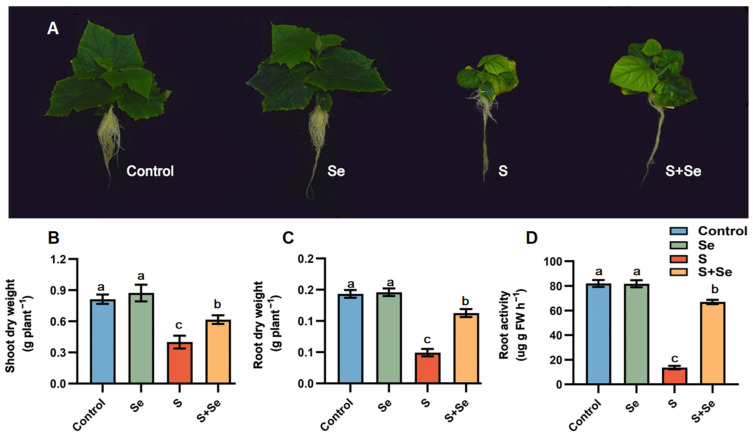
Effects of sodium selenite (Na_2_SeO_3_, Se) on cucumber (*Cucumis sativus* L.)seedling growth under NaHCO_3_ stress. (**A**) Phenotype after 8 days of treatment; (**B**) shoot dry weight; (**C**) root dry weight; (**D**) root activity. Treatments: Control, Hoagland solution; Se, Hoagland + Na_2_SeO_3_; S, Hoagland + NaHCO_3_; S + Se, Hoagland + NaHCO_3_ + Na_2_SeO_3_. Values are presented as the mean ± SD (*n* = 3). Different lowercase letters indicate significant differences among treatments at *p* < 0.05 according to Duncan’s multiple range test.

As shown in Figure 1, NaHCO_3_ treatment markedly inhibited the growth of cucumber seedlings, leading to chlorosis and wilting of leaves (Figure 1A), as well as significant reductions in shoot biomass (Figure 1B), root biomass (Figure 1C), and triphenyltetrazolium chloride (TTC)-reduction-based root metabolic activity (Figure 1D). Application of 2 μmol·L^−1^ sodium selenite (Se) alleviated these negative effects, mitigating leaf chlorosis and improving biomass accumulation and root metabolic activity under NaHCO_3_ stress.

**Figure 2:**
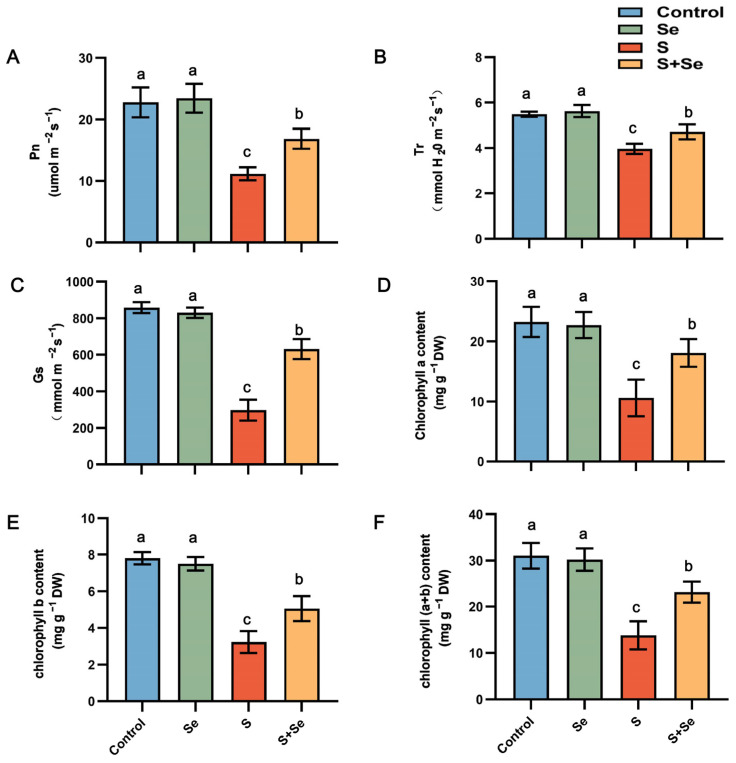
Effects of exogenous sodium selenite (Na_2_SeO_3_, Se) on net photosynthetic rate (**A**), transpiration rate (**B**), stomatal conductance (**C**), chlorophyll a content (**D**), chlorophyll b content (**E**), and total chlorophyll (a + b) content (**F**) of cucumber seedlings under NaHCO_3_ stress. Treatments: Control, Hoagland solution; Se, Hoagland + Na_2_SeO_3_; S, Hoagland + NaHCO_3_; S + Se, Hoagland + NaHCO_3_ + Na_2_SeO_3_. Values are presented as the mean ± SD (*n* = 3). Different lowercase letters indicate significant differences among treatments at *p* < 0.05 according to Duncan’s multiple range test.

As illustrated in Figure 2, NaHCO_3_ treatment markedly suppressed photosynthetic traits, including net photosynthetic rate (Pn), transpiration rate (Tr), and stomatal conductance (Gs), along with declines in chlorophyll a and b levels. Application of Se effectively mitigated these negative influences by enhancing Pn, Tr, and Gs while restoring chlorophyll a, chlorophyll b, and overall chlorophyll accumulation under stress. These findings indicate that Se supplementation contributes to sustaining photosynthetic efficiency and pigment integrity in cucumber exposed to alkaline stress.

As shown in Table 1, NaHCO_3_ stress markedly reduced Fv/Fm, Fv′/Fm′, ΦPSII, qP, and Ex compared with the control, indicating severe impairment of PSII photochemical efficiency and excitation energy flux. Application of Se under normal growth conditions did not significantly alter these parameters relative to the control. However, Se supplementation under NaHCO_3_ stress significantly improved Fv/Fm, Fv′/Fm′, ΦPSII, qP, and Ex compared with NaHCO_3_ treatment alone, although the values did not fully recover to the control level. These results suggest that Se effectively alleviates PSII photoinhibition and partially restores photosynthetic efficiency in cucumber seedlings exposed to alkaline stress.

### 2.2. Exogenous Se Enhances Antioxidant Capacity and Mitigates Oxidative Damage Induced by Alkaline (NaHCO_3_) Stress

**Figure 3:**
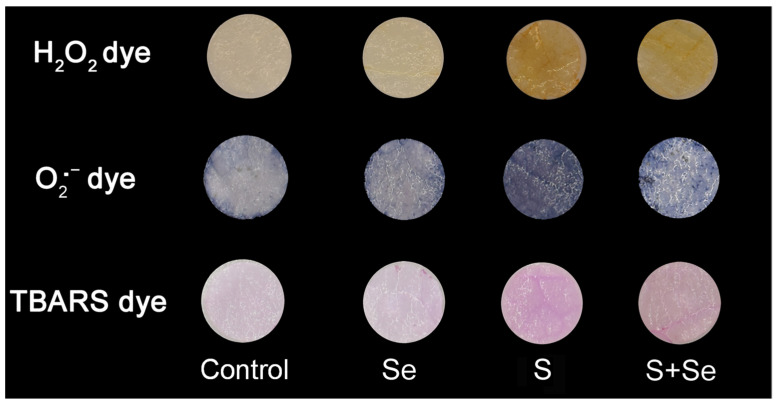
Effects of exogenous sodium selenite (Na_2_SeO_3_, Se) on histochemical staining of superoxide anion (O_2_·^−^), hydrogen peroxide (H_2_O_2_), and thiobarbituric acid reactive substances (TBARS) in cucumber leaves under NaHCO_3_ stress. Histochemical staining was performed with nitro blue tetrazolium (NBT) for O_2_·^−^, 3,3′-diaminobenzidine (DAB) for H_2_O_2_, and TBARS staining for lipid peroxidation, mainly reflecting malondialdehyde (MDA) accumulation. Treatments: Control, Hoagland solution; Se, Hoagland + Na_2_SeO_3_; S, Hoagland + NaHCO_3_; S + Se, Hoagland + NaHCO_3_ + Na_2_SeO_3_.

As shown in Figure 3, under NaHCO_3_ stress, NBT staining for O_2_·^−^ and DAB staining for H_2_O_2_ indicated that NaHCO_3_ stress markedly increased the accumulation of both ROSs, accompanied by enhanced TBARS deposition, reflecting elevated lipid peroxidation. In contrast, Se treatment significantly reduced the accumulation of O_2_·^−^, H_2_O_2_, and TBARS under NaHCO_3_ stress, as evidenced by lighter staining. These results demonstrate that exogenous Se effectively mitigates oxidative damage and suppresses malondialdehyde (MDA) accumulation in cucumber seedlings subjected to alkaline stress.

**Figure 4:**
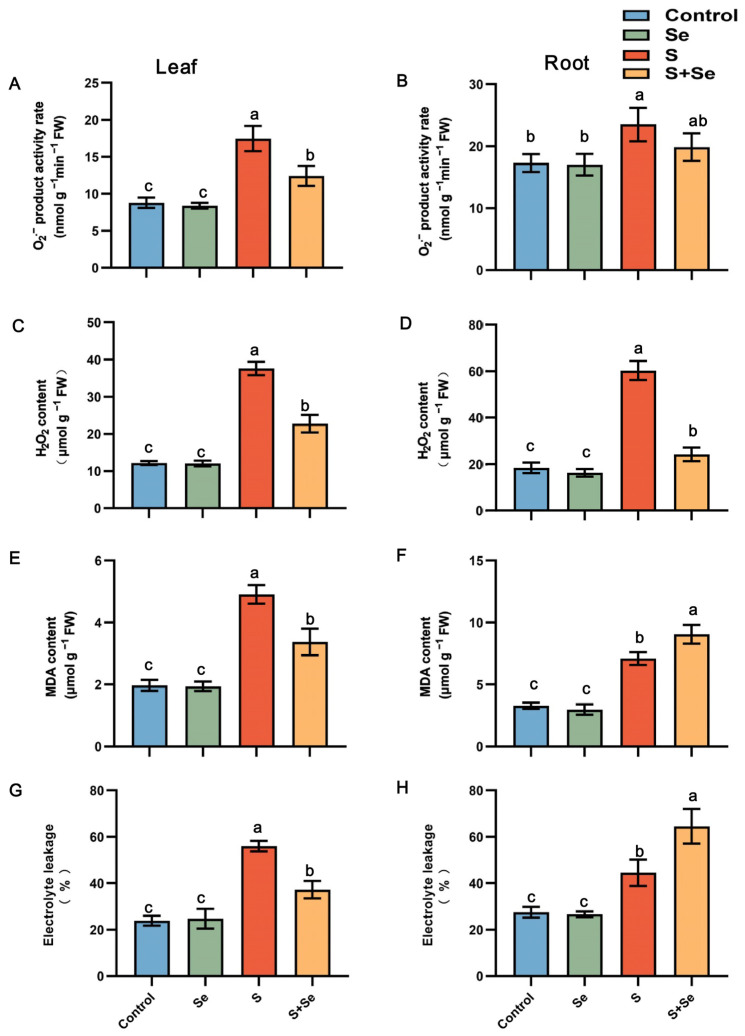
Effects of exogenous sodium selenite (Na_2_SeO_3_, Se) on oxidative damage in cucumber seedlings under NaHCO_3_ stress. (**A**,**B**) O_2_·^−^ content in leaves and roots; (**C**,**D**) H_2_O_2_ production rate in leaves and roots; (**E**,**F**) MDA content in leaves and roots; (**G**,**H**) electrolyte leakage in leaves and roots. Treatments: Control, Hoagland solution; Se, Hoagland + Na_2_SeO_3_; S, Hoagland + NaHCO_3_; S + Se, Hoagland + NaHCO_3_ + Na_2_SeO_3_. Values are presented as the mean ± SD (*n* = 3). Different lowercase letters indicate significant differences among treatments at *p* < 0.05 according to Duncan’s multiple range test.

As shown in Figure 4, exogenous Se had little effect on ROS accumulation, MDA content, or electrolyte leakage in cucumber seedlings under normal growth conditions. NaHCO_3_ stress markedly increased the O_2_· production rate (Figure 4A,B), H_2_O_2_ content (Figure 4C,D), MDA levels (Figure 4E,F), and electrolyte leakage (Figure 4G,H) in both leaves and roots. In contrast, Se supplementation under NaHCO_3_ stress significantly reduced ROS accumulation in both leaves and roots. Se also markedly decreased MDA content and electrolyte leakage in leaves, whereas the corresponding changes in roots were less evident.

**Figure 5:**
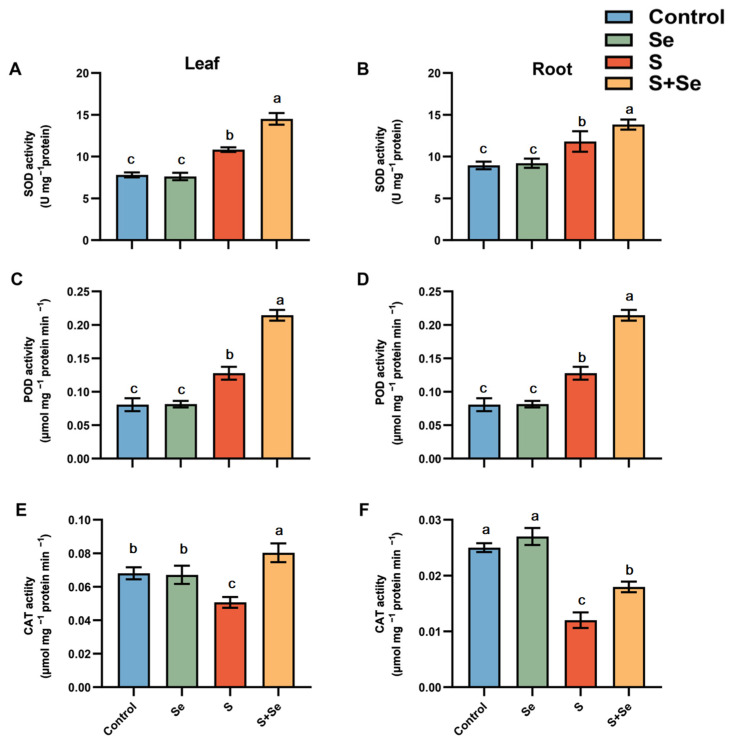
Effects of exogenous sodium selenite (Na_2_SeO_3_, Se) on antioxidant enzyme activities in cucumber seedlings under NaHCO_3_ stress. (**A**,**B**) superoxide dismutase (SOD) activity in leaves and roots; (**C**,**D**) peroxidase (POD) activity in leaves and roots; (**E**,**F**) catalase (CAT) activity in leaves and roots. Treatments: Control, Hoagland solution; Se, Hoagland + Na_2_SeO_3_; S, Hoagland + NaHCO_3_; S + Se, Hoagland + NaHCO_3_ + Na_2_SeO_3_. Values are presented as the mean ± SD (*n* = 3). Different lowercase letters indicate significant differences among treatments at *p* < 0.05 according to Duncan’s multiple range test.

Under control conditions, Se addition had minimal influence on SOD, POD, and CAT in either leaves or roots. NaHCO_3_ stress enhanced SOD and POD but suppressed CAT activity. When Se was supplied under NaHCO_3_ stress, the activities of SOD, POD, and CAT in both leaves and roots were markedly elevated, suggesting that Se strengthens the antioxidant defense system of cucumber seedlings facing alkaline stress (Figure 5).

**Figure 6:**
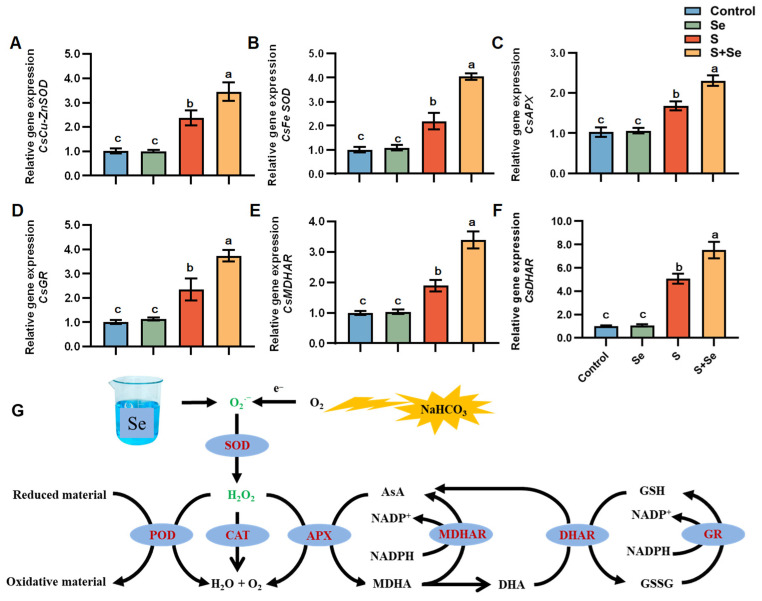
Effects of exogenous sodium selenite (Na_2_SeO_3_, Se) on the expression of antioxidant-related genes in cucumber roots under NaHCO_3_ stress. (**A**) Cu/Zn-superoxide dismutase (*CsCu*/*ZnSOD*) expression; (**B**) Fe-superoxide dismutase (*CsFeSOD*) expression; (**C**) Ascorbate peroxidase (*CsAPX*) expression; (**D**) Dehydroascorbate reductase (*CsDHAR*) expression; (**E**) Monodehydroascorbate reductase (*CsMDHAR*) expression; (**F**) Glutathione reductase (*CsGR*) expression; (**G**) Schematic representation of the Se-induced antioxidant network under stress, including the AsA–GSH cycle. Green text indicates factors downregulated by Se under NaHCO_3_ stress, while red text denotes factors upregulated by Se. Treatments: Control, Hoagland solution; Se, Hoagland + Na_2_SeO_3_; S, Hoagland + NaHCO_3_; S + Se, Hoagland + NaHCO_3_ + Na_2_SeO_3_. Gene expression was determined after 24 h of treatment. Values are presented as the mean ± SD (*n* = 3). Different lowercase letters indicate significant differences among treatments at *p* < 0.05 according to Duncan’s multiple range test.

As illustrated in Figure 6, NaHCO_3_ treatment strongly upregulated the expression of Cu/Zn-superoxide dismutase (*CsCu*/*ZnSOD*), Fe-superoxide dismutase (*CsFeSOD*), Ascorbate peroxidase (*CsAPX*), Dehydroascorbate reductase (*CsDHAR*), Monodehydroascorbate reductase (*CsMDHAR*), and Glutathione reductase (*CsGR*) compared with the control. Supplementation with Se further boosted the transcription of these genes, with *CsGR* and *CsDHAR* showing the greatest induction. In contrast, Se alone produced only minor changes. These findings suggest that Se enhances antioxidant defense at the transcriptional level, particularly within the AsA–GSH cycle, thereby improving root tolerance to oxidative damage under alkaline stress.

### 2.3. Exogenous Se Maintains Ion Homeostasis Under NaHCO_3_ Stress

**Figure 7:**
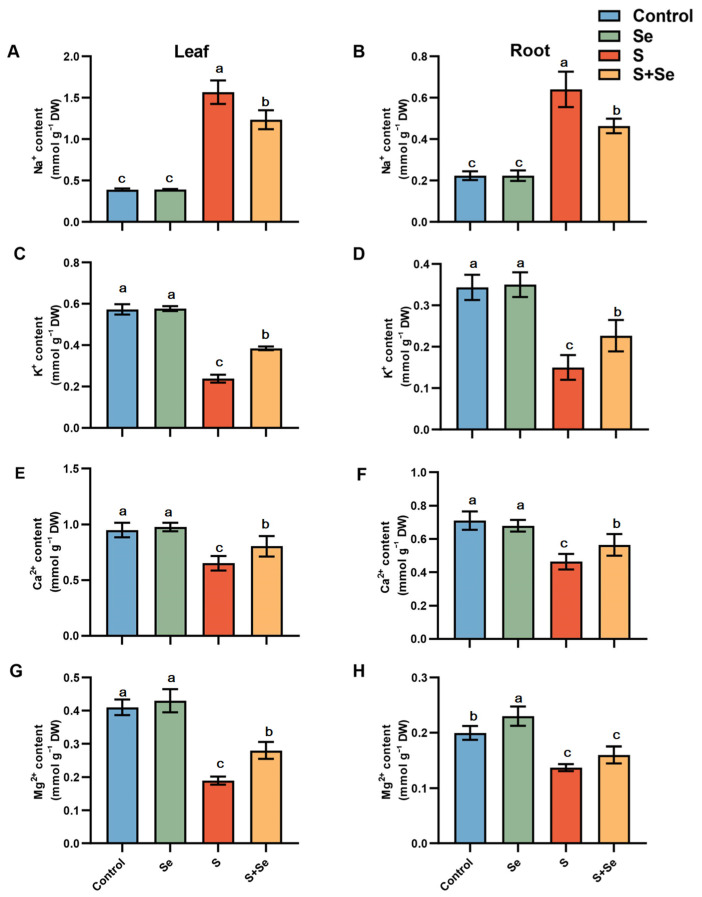
Effects of sodium selenite (Na_2_SeO_3_, Se) on ion concentrations in cucumber seedlings under NaHCO_3_ stress. (**A**,**B**) Na^+^ levels in leaves and roots; (**C**,**D**) K^+^ levels in leaves and roots; (**E**,**F**) Ca^2+^ levels in leaves and roots; (**G**,**H**) Mg^2+^ levels in leaves and roots. Treatments: Control, Hoagland solution; Se, Hoagland + Na_2_SeO_3_; S, Hoagland + NaHCO_3_; S + Se, Hoagland + NaHCO_3_ + Na_2_SeO_3_. Values are presented as the mean ± SD (*n* = 3). Different lowercase letters indicate significant differences among treatments at *p* < 0.05 according to Duncan’s multiple range test.

**Figure 8:**
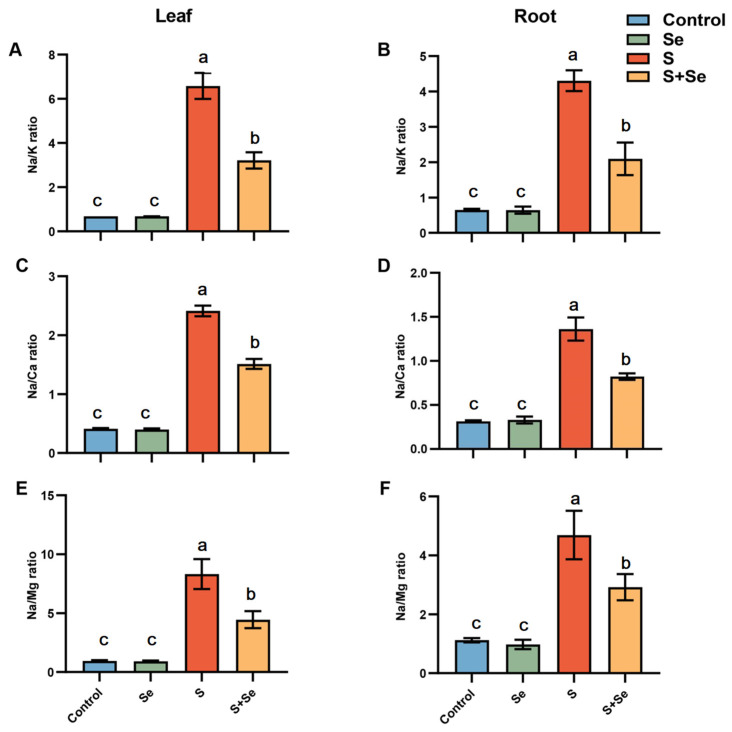
Effects of exogenous sodium selenite (Na_2_SeO_3_, Se) on ion ratios in cucumber seedlings under NaHCO_3_ stress. (**A**,**B**) Na^+^/K^+^ ratio in leaves and roots; (**C**,**D**) Na^+^/Ca^2+^ ratio in leaves and roots; (**E**,**F**) Na^+^/Mg^2+^ ratio in leaves and roots. Treatments: Control, Hoagland solution; Se, Hoagland + Na_2_SeO_3_; S, Hoagland + NaHCO_3_; S + Se, Hoagland + NaHCO_3_ + Na_2_SeO_3_. Values are presented as the mean ± SD (*n* = 3). Different lowercase letters indicate significant differences among treatments at *p* < 0.05 according to Duncan’s multiple range test.

As shown in Figure 7 and Figure 8, NaHCO_3_ stress markedly increased Na^+^ accumulation in both leaves and roots while significantly reducing K^+^, Ca^2+^, and Mg^2+^ contents. Consequently, the Na^+^/K^+^, Na^+^/Ca^2+^, and Na^+^/Mg^2+^ ratios were dramatically elevated, reflecting ionic imbalance under alkaline conditions. In contrast, exogenous Se treatment effectively decreased Na^+^ accumulation, promoted K^+^, Ca^2+^, and Mg^2+^ uptake, and lowered Na^+^/K^+^, Na^+^/Ca^2+^, and Na^+^/Mg^2+^ ratios compared with NaHCO_3_ stress alone. These results suggest that Se alleviates Na^+^ toxicity and contributes to maintaining ionic homeostasis in cucumber seedlings exposed to alkaline stress.

**Figure 9:**
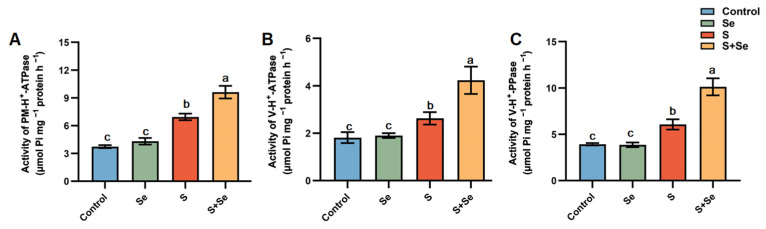
Effects of exogenous sodium selenite (Na_2_SeO_3_, Se) on the activities of membrane-associated proton pumps in cucumber roots under NaHCO_3_ stress. (**A**) Plasma membrane H^+^-ATPase activity; (**B**) tonoplast H^+^-ATPase; (**C**) tonoplast H^+^-PPase. Treatments: Control, Hoagland solution; Se, Hoagland + Na_2_SeO_3_; S, Hoagland + NaHCO_3_; S + Se, Hoagland + NaHCO_3_ + Na_2_SeO_3_. Values are presented as the mean ± SD (*n* = 3). Different lowercase letters indicate significant differences among treatments at *p* < 0.05 according to Duncan’s multiple range test.

As shown in Figure 9, NaHCO_3_ stress elevated the activity of plasma membrane H^+^-ATPase, tonoplast H^+^-ATPase, and H^+^-PPase in cucumber roots relative to the control. The addition of Se further promoted these proton pump activities. Such stimulation of H^+^-ATPase and H^+^-PPase by Se facilitates Na^+^ extrusion at the plasma membrane and sequestration into vacuoles, thereby supporting ionic balance under alkaline stress.

**Figure 10:**
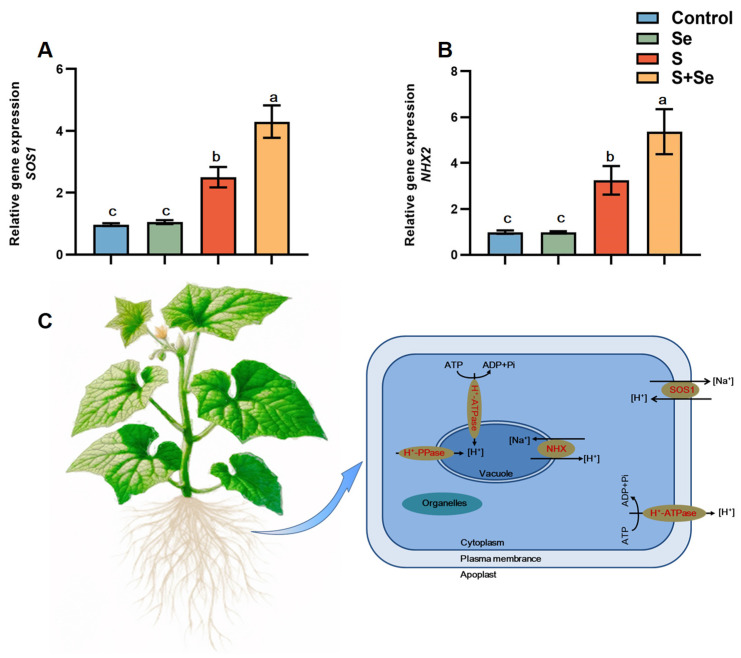
Effects of exogenous sodium selenite (Na_2_SeO_3_, Se) on the expression of ion transport-related genes and a schematic model of Na^+^ regulation in cucumber roots under NaHCO_3_ stress. (**A**) Salt Overly Sensitive 1 (*SOS1*) expression; (**B**) Na^+^/H^+^ exchanger 2 (*NHX2*) expression; (**C**) proposed model illustrating Na^+^ transport and vacuolar sequestration. Exogenous Se promoted the expression of *SOS1* and *NHX2* and enhanced the activity of vacuolar H^+^-ATPase, H^+^-PPase, and plasma membrane H^+^-ATPase, thereby supporting Na^+^ extrusion and vacuolar sequestration. These adjustments reduced Na^+^ toxicity and contributed to maintaining ion homeostasis under alkaline stress. Proteins stimulated by Se are indicated in red in the diagram. Treatments: Control, Hoagland solution; Se, Hoagland + Na_2_SeO_3_; S, Hoagland + NaHCO_3_; S + Se, Hoagland + NaHCO_3_ + Na_2_SeO_3_. Gene expression was determined after 24 h of treatment. Values are presented as the mean ± SD (*n* = 3). Different lowercase letters indicate significant differences among treatments at *p* < 0.05 according to Duncan’s multiple range test.

As shown in Figure 10, NaHCO_3_ stress markedly elevated the transcript abundance of Salt Overly Sensitive 1 and Na^+^/H^+^ exchanger 2 (*NHX2*) expression, two genes closely linked to Na^+^ detoxification via the SOS pathway. Application of exogenous selenium further reinforced their induction under stress conditions. These results suggest that Se enhances Na^+^ detoxification in cucumber roots through modulation of the SOS signaling cascade. The schematic representation (Figure 10) indicates that Se not only stimulates *SOS1* and *NHX2* expression but also augments the activities of vacuolar H^+^-ATPase, H^+^-PPase, and plasma membrane H^+^-ATPase. This combined effect promotes Na^+^ extrusion and vacuolar sequestration, thereby alleviating sodium toxicity and supporting ion balance under alkaline stress.

### 2.4. Exogenous Se Enhances TCA Cycle Enzyme Activities and Organic Acid Accumulation Under Alkaline (NaHCO_3_) Stress

**Figure 11:**
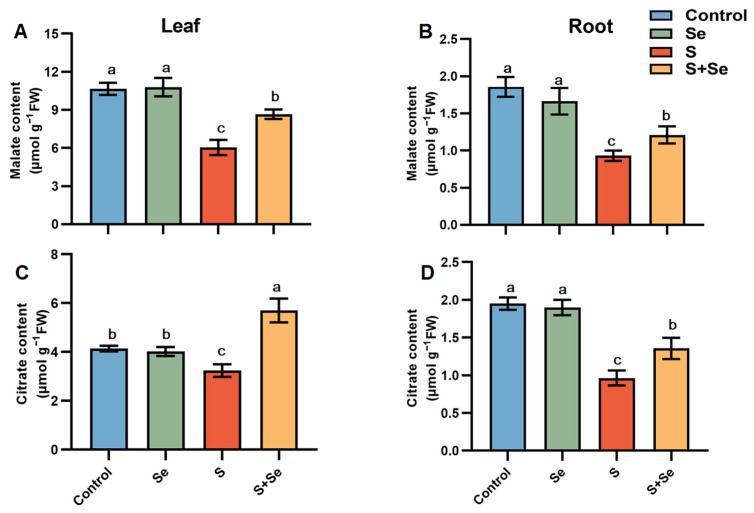
Effects of exogenous sodium selenite (Na_2_SeO_3_, Se) on organic acid contents in cucumber seedlings under NaHCO_3_ stress. (**A**,**B**) citrate content in leaves and roots; (**C**,**D**) malate content in leaves and roots. Treatments: Control, Hoagland solution; Se, Hoagland + Na_2_SeO_3_; S, Hoagland + NaHCO_3_; S + Se, Hoagland + NaHCO_3_ + Na_2_SeO_3_. Values are presented as the mean ± SD (*n* = 3). Different lowercase letters indicate significant differences among treatments at *p* < 0.05 according to Duncan’s multiple range test.

As shown in Figure 11, NaHCO_3_ stress significantly reduced citrate and malate contents in cucumber leaves and roots compared with the control treatment. Under alkaline stress conditions, exogenous Se application partially alleviated the decline in these organic acids, with a more pronounced effect observed for citrate accumulation. These results suggest that Se may help maintain organic acid homeostasis under NaHCO_3_ stress, thereby contributing to the alleviation of high-pH-induced damage in cucumber seedlings.

**Figure 12:**
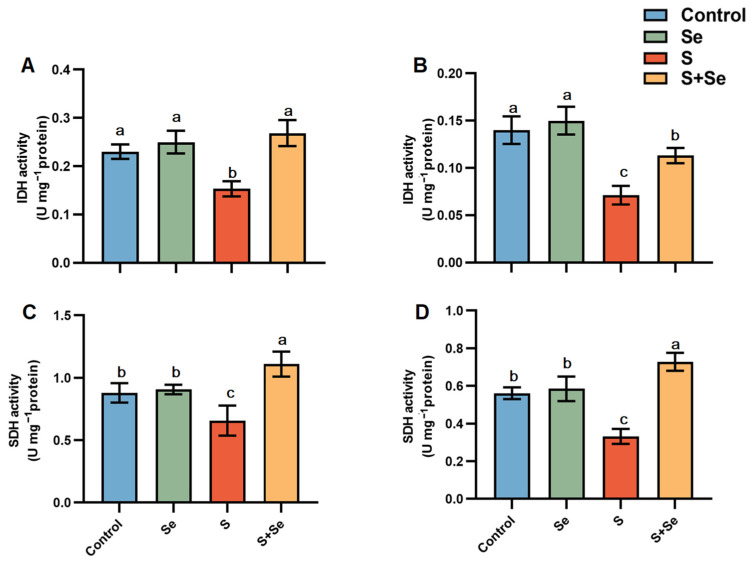
Effects of exogenous sodium selenite (Na_2_SeO_3_, Se) on the activities of tricarboxylic acid (TCA) cycle enzymes in cucumber seedlings under NaHCO_3_ stress. (**A**,**B**) isocitrate dehydrogenase (IDH) activity in leaves and roots; (**C**,**D**) succinate dehydrogenase (SDH) activity in leaves and roots. Treatments: Control, Hoagland solution; Se, Hoagland + Na_2_SeO_3_; S, Hoagland + NaHCO_3_; S + Se, Hoagland + NaHCO_3_ + Na_2_SeO_3_. Values are presented as the mean ± SD (*n* = 3). Different lowercase letters indicate significant differences among treatments at *p* < 0.05 according to Duncan’s multiple range test.

As illustrated in Figure 12, NaHCO_3_ stress reduced the activities of IDH and SDH in both leaves and roots. Notably, Se supplementation under alkaline conditions significantly enhanced the activities of these enzymes in both tissues, thereby improving overall TCA cycle functionality. These findings suggest that Se fortifies TCA cycle activity, thereby sustaining energy metabolism and enhancing cucumber tolerance to high-pH stress.

### 2.5. Exogenous Se Promotes Osmotic Adjustment by Regulating Compatible Solutes and Aquaporin Expression Under Alkaline (NaHCO_3_) Stress

**Figure 13:**
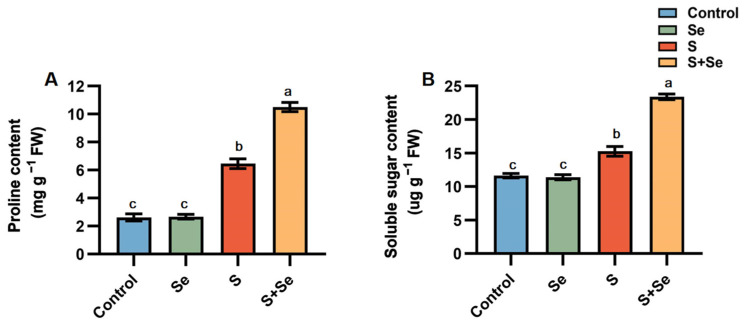
Influence of exogenous sodium selenite (Na_2_SeO_3_, Se) on osmolyte accumulation in cucumber leaves exposed to NaHCO_3_ stress. (**A**) Proline levels; (**B**) soluble sugar levels. Treatments: Control, Hoagland solution; Se, Hoagland + Na_2_SeO_3_; S, Hoagland + NaHCO_3_; S + Se, Hoagland + NaHCO_3_ + Na_2_SeO_3_. Values are presented as the mean ± SD (*n* = 3). Different lowercase letters indicate significant differences among treatments at *p* < 0.05 according to Duncan’s multiple range test.

As shown in Figure 13, NaHCO_3_ stress significantly increased the accumulation of soluble proline (A) and sugars (B) in cucumber leaves compared with the control. Se supplementation further enhanced the contents of these osmolytes under stress conditions, suggesting that Se promotes osmolyte accumulation to improve osmotic adjustment.

**Figure 14:**
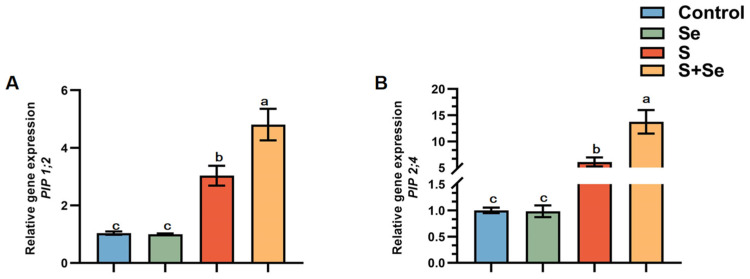
Effect of exogenous sodium selenite (Na_2_SeO_3_, Se) on aquaporin gene expression in cucumber roots subjected to NaHCO_3_ stress. (**A**) Plasma membrane intrinsic protein 1;2 (*PIP1;2*) expression; (**B**) Plasma membrane intrinsic protein 2;4 (*PIP2;4*) expression. Treatments: Control, Hoagland solution; Se, Hoagland + Na_2_SeO_3_; S, Hoagland + NaHCO_3_; S + Se, Hoagland + NaHCO_3_ + Na_2_SeO_3_. Gene expression was determined after 24 h of treatment. Values are presented as the mean ± SD (*n* = 3). Different lowercase letters indicate significant differences among treatments at *p* < 0.05 according to Duncan’s multiple range test.

In addition, Figure 14 shows that NaHCO_3_ stress induced the expression of the aquaporin genes plasma membrane intrinsic protein 1;2 (*PIP1;2*) (A) and plasma membrane intrinsic protein 2;4 (*PIP2;4*) in cucumber roots. Se treatment further upregulated their expression levels compared with NaHCO_3_ alone, indicating that Se facilitates water transport by regulating aquaporin-mediated pathways.

Together, these results suggest that Se enhances osmotic adjustment under alkaline stress through dual regulation: increasing osmolyte accumulation in leaves and promoting aquaporin expression in roots, thereby contributing to cellular water balance and stress tolerance.

### 2.6. Exogenous Se Restores Salicylic Acid Biosynthesis Under Alkaline (NaHCO_3_) Stress

**Figure 15:**
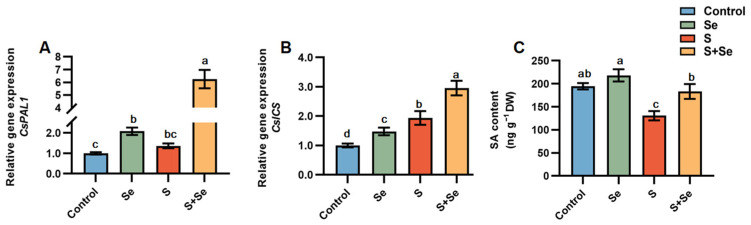
Impact of exogenous sodium selenite (Na_2_SeO_3_, Se) on salicylic acid (SA) metabolism and the expression of SA biosynthesis-related genes in cucumber leaves subjected to NaHCO_3_ stress. (**A**) Isochorismate synthase (*CsICS*) expression; (**B**) Phenylalanine ammonia-lyase (*CsPAL1*) expression; (**C**) SA content. Treatments: Control, Hoagland solution; Se, Hoagland + Na_2_SeO_3_; S, Hoagland + NaHCO_3_; S + Se, Hoagland + NaHCO_3_ + Na_2_SeO_3_. Gene expression was determined after 24 h of treatment. Values are presented as the mean ± SD (*n* = 3). Different lowercase letters indicate significant differences among treatments at *p* < 0.05 according to Duncan’s multiple range test.

Under normal growth conditions, exogenous Se treatment slightly increased the expression levels of phenylalanine ammonia-lyase (*CsPAL1*) and isochorismate synthase (*CsICS*), accompanied by a moderate increase in leaf salicylic acid (SA) content (Figure 15A–C). After 24 h of NaHCO_3_ stress, the transcript level of CsPAL1 was upregulated, whereas CsICS expression was reduced. However, Se supplementation under NaHCO_3_ stress significantly enhanced the expression levels of both CsICS and CsPAL1. Consistently, after 8 days of treatment, NaHCO_3_ stress significantly decreased SA accumulation in cucumber leaves, while exogenous Se application partially restored leaf SA content.

## 3. Discussion

NaHCO_3_ stress markedly inhibited cucumber seedling growth, as evidenced by reductions in both shoot and root biomass as well as decreased root activity. These observations are in line with previous findings on alkaline salt stress in crops such as cucumber, tomato, and rice, which demonstrated that alkaline stress severely constrains plant growth through a combination of Na^+^ toxicity, elevated pH, nutrient precipitation, osmotic imbalance, and oxidative stress [6,42]. In the present study, exogenous sodium selenite (Na_2_SeO_3_) significantly alleviated the growth inhibition caused by NaHCO_3_, leading to increased biomass in both shoots and roots and improved root activity. Comparable findings have been observed in other crops: Se application promoted growth and dry matter accumulation in buckwheat [43], enhanced seedling growth and photosynthetic performance in tomato exposed to salt stress [44], and improved root development and plant quality in lettuce [45]. Collectively, these results indicate that Se, at appropriate concentrations, functions as a beneficial element under saline–alkali stress, improving plant growth and vigor through multiple mechanisms, including reinforcement of antioxidant defense, maintenance of ion homeostasis, and enhancement of osmotic regulation.

NaHCO_3_ stress significantly impaired photosynthetic performance in cucumber seedlings. Relative to the control, NaHCO_3_ exposure led to a pronounced decline in net photosynthetic rate (Pn), transpiration rate (Tr), stomatal conductance (Gs), as well as chlorophyll a, chlorophyll b, and total chlorophyll (Chl a + b). The decline in chlorophyll levels indicates accelerated pigment degradation, while the reduction in Pn reflects both stomatal closure and non-stomatal limitations, including damage to photosystem II (PSII) [46,47,48]. These findings suggest that salt stress decreases photosynthetic efficiency through both impaired gas exchange and reduced light-harvesting capacity, thereby limiting biomass accumulation. Chlorophyll fluorescence parameters further confirmed photosynthetic impairment from a photochemical perspective: decreases in Fv/Fm, Fv′/Fm′, and ΦPSII indicated impaired PSII activity and electron transport; reductions in qP reflected diminished efficiency of PSII open reaction centers; and lower Ex suggested insufficient excitation energy for photochemical reactions [46,47,48]. These results are consistent with previous reports showing that alkaline stress disrupts photosynthetic performance and energy balance in crops such as tomato and rice [44,46,48,49].

Exogenous sodium selenite (Na_2_SeO_3_) partially alleviated these adverse effects. Compared with NaHCO_3_ treatment alone, Se-treated plants showed higher Pn, Gs, and Tr, along with restored chlorophyll a, chlorophyll b, and Chl a + b contents. Simultaneously, Fv/Fm, Fv′/Fm′, and ΦPSII were significantly increased, while qP and Ex also improved, indicating that Se helped stabilize PSII and enhance the photochemical utilization of light energy [46,47,48]. This protective effect is likely related to selenium-mediated enhancement of antioxidant enzyme activity, ROS scavenging, and maintenance of ionic homeostasis and osmotic balance, thereby preventing further chloroplast damage [46,47,48]. Similar findings have been documented in tomato, where selenium application improved Fv/Fm and photosynthetic traits under salinity while enhancing CO_2_ assimilation and leaf physiological performance [44]. In wheat, application of low levels of Na_2_SeO_4_ or Na_2_SeO_3_ reduced declines in fluorescence indices under salt stress, decreased ROS and membrane lipid peroxidation, and maintained relatively high photosynthetic efficiency [50]. Collectively, these findings suggest that the beneficial effects of Se on photosynthesis are closely associated with coordinated protection of chloroplast structure, maintenance of chlorophyll stability, and alleviation of ROS-induced photochemical damage. Since chlorophyll biosynthesis and PSII function are highly dependent on Mg^2+^ availability and membrane integrity, the Se-mediated improvement in ionic homeostasis and antioxidant capacity may jointly contribute to the restoration of photosynthetic efficiency under alkaline stress.

Abiotic stress is often accompanied by excessive accumulation of reactive oxygen species (ROS), which are major contributors to plant cell damage [51]. In this study, NaHCO_3_ treatment significantly increased O_2_·^−^ and H_2_O_2_ contents, along with higher MDA levels and electrolyte leakage, indicating that ROS accumulation accelerated membrane lipid peroxidation and impaired cell membrane stability [41,51]. Supplemental sodium selenite markedly alleviated this oxidative damage in cucumber seedlings. Se-treated plants exhibited lower ROS accumulation in both leaves and roots and markedly reduced MDA accumulation in leaves, indicating that Se effectively alleviated oxidative damage under alkaline stress, consistent with previous findings [52,53]. This protective effect is closely associated with Se-induced enhancement of antioxidant enzyme activities. Interestingly, although NaHCO_3_ stress increased SOD and POD activities, CAT activity was significantly reduced. This response suggests that severe alkaline stress may impair CAT activity because excessive ROS accumulation exceeds its detoxification capacity, whereas SOD and POD are rapidly induced as compensatory antioxidant enzymes. Selenium application effectively restored CAT activity while further enhancing SOD and POD activities, thereby improving the overall ROS scavenging capacity and alleviating oxidative damage.

Moreover, Se treatment also upregulated the expression of antioxidant-related genes, including *CsCu*/*ZnSOD*, *CsFeSOD*, *CsAPX*, *CsMDHAR*, *CsDHAR*, and *CsGR*, suggesting that Se may enhance the AsA-GSH cycle under alkaline stress [52,54]. The coordinated upregulation of these redox-related genes implies a strengthened capacity for H_2_O_2_ detoxification and cellular redox regulation, thereby providing an additional protective barrier against oxidative damage [52,54]. The simultaneous enhancement of antioxidant enzyme activities and antioxidant-related gene expression indicates that Se establishes a more comprehensive and coordinated antioxidant defense network, consistent with studies in *Setaria italica* and *Phaseolus vulgaris* and recent reviews on mechanisms of plant stress tolerance [55,56,57]. By enhancing ROS scavenging capacity, Se contributes to redox homeostasis and reduces chloroplast damage, thereby indirectly supporting photosynthetic stability under alkaline stress [53,56]. These results further suggest that the protective role of Se is not limited to stimulating antioxidant enzyme activities but also involves the regulation of antioxidant-related gene expression. By maintaining redox homeostasis and reducing membrane lipid peroxidation, Se likely protects chloroplast membranes and the photosynthetic apparatus from oxidative injury, thereby contributing to chlorophyll preservation, improved PSII activity, and enhanced carbon assimilation under NaHCO_3_ stress [52,53,56].

Saline–alkali stress often disrupts plant ionic equilibrium by promoting Na^+^ accumulation while inhibiting the uptake of K^+^, Ca^2+^, and Mg^2+^ [1,58]. In this study, NaHCO_3_ treatment significantly increased Na^+^ content in cucumber leaves and roots while markedly reducing K^+^, Ca^2+^, and Mg^2+^ levels. This resulted in substantial increases in the Na^+^/K^+^, Na^+^/Ca^2+^, and Na^+^/Mg^2+^ ratios, indicating severe disruption of ion homeostasis. Supplementation with exogenous sodium selenite (Na_2_SeO_3_, Se) effectively alleviated this imbalance by reducing Na^+^ accumulation and promoting the uptake of K^+^, Ca^2+^, and Mg^2+^, thereby significantly improving ion ratios. The effect was most pronounced for K^+^ homeostasis, as Na^+^ and K^+^ compete for entry through non-selective cation channels, with high Na^+^ frequently inhibiting K^+^ uptake [58,59]. K^+^ deficiency is often associated with growth inhibition and reduced enzyme activity, and the enhancement of K^+^ retention capacity by Se may represent a key mechanism underlying its stress-alleviating effects [40,59].

Ca^2+^ and Mg^2+^ also play vital roles. Ca^2+^ is essential for stabilizing cell membranes and cell walls and acts as a secondary messenger in stress signaling [60], whereas Mg^2+^ is the central component of chlorophyll and an activator of Rubisco, directly influencing photosynthetic efficiency [61,62]. Under Se treatment, Ca^2+^ and Mg^2+^ homeostasis was better maintained, contributing to the stabilization of cellular structures and supporting normal photosynthetic function. Since Mg^2+^ constitutes the central ion of the chlorophyll molecule, the maintenance of Mg^2+^ homeostasis under Se treatment may also have contributed to the preservation of chlorophyll content and the recovery of PSII activity observed in this study. Mechanistically, Se also enhanced the activity of membrane-associated proton pumps. Plasma membrane H^+^-ATPase establishes a proton gradient using ATP hydrolysis, thereby driving Na^+^ efflux across the membrane, while vacuolar H^+^-ATPase and H^+^ -PPase generate a proton motive force within vacuoles, enabling Na^+^ sequestration through the Na^+^/H^+^ antiporter. H^+^ -ATPase relies on ATP, whereas H^+^-PPase utilizes pyrophosphate, and their synergistic activity is crucial for Na^+^ compartmentalization. In this study, Se treatment significantly increased the activities of these proton pumps, thereby providing the proton motive force required for Na^+^ detoxification. Gene expression analysis further supported this finding: NaHCO_3_ stress induced the expression of *SOS1* and *NHX2*, and Se further enhanced their transcriptional levels, suggesting that Se not only enhances Na^+^ transport capacity but also activates molecular pathways related to Na^+^ detoxification.

Similar regulatory effects have been reported in other crops. In cucumber, Amerian et al. (2024) observed that Se treatment improved ion tolerance under salt stress by reducing membrane conductance and ion leakage [20]. In tomato, Zhang et al. (2023) found that exogenous Se restored ion homeostasis, reduced the Na^+^/K^+^ ratio, and simultaneously enhanced biomass and photosynthetic efficiency [63]. In summary, Se mitigates ion imbalance under NaHCO_3_ stress through multiple pathways: decreasing Na^+^ accumulation, promoting K^+^, Ca^2+^, and Mg^2+^ uptake, and enhancing H^+^-ATPase and H^+^-PPase activity to facilitate Na^+^ efflux and vacuolar compartmentalization; concurrently upregulating *SOS1* and *NHX2* to strengthen Na^+^ detoxification. Collectively, these processes maintain ionic homeostasis and enhance cucumber resistance to alkaline stress.

Through glycolysis, plants generate pyruvate, which subsequently enters the tricarboxylic acid (TCA) cycle and undergoes a series of oxidative reactions, ultimately producing CO_2_, H_2_O, and ATP to sustain essential cellular functions [64]. The TCA cycle is the most energy-efficient respiratory pathway and serves as the primary route by which plants derive energy from carbohydrates [64,65]. Previous studies have shown that saline–alkali stress inhibits TCA cycle activity [66]. In the present study, NaHCO_3_ stress significantly decreased the activities of isocitrate dehydrogenase (IDH) and succinate dehydrogenase (SDH) in cucumber leaves and roots, whereas exogenous sodium selenite (Na_2_SeO_3_, Se) markedly enhanced the activities of these two key enzymes. These results suggest that Se alleviates alkaline stress-induced inhibition of the TCA cycle, thereby supporting cellular energy metabolism. Since excessive ROS accumulation can impair TCA cycle enzymes, the recovery of IDH and SDH activities may be associated with the enhanced antioxidant capacity and ROS scavenging ability induced by Se.

Regarding organic acid metabolism, previous studies have reported that saline–alkali stress may promote organic acid accumulation under moderate stress but reduce it under severe stress [66]. In the present study, NaHCO_3_ treatment decreased citric and malic acid contents in cucumber leaves and roots, whereas Se application significantly promoted their accumulation. Consistently, SeNPs combined with biochar improved wheat performance by enhancing K^+^, Ca^2+^, and Mg^2+^ uptake and promoting organic acid metabolism [67]. Organic acids such as citrate and malate may contribute to ionic balance and osmotic adjustment under saline–alkali stress, which may partially explain the improved stress tolerance observed in Se-treated plants. However, the present study did not directly evaluate cation chelation or tissue/rhizosphere pH; therefore, the precise contribution of organic acids to ion homeostasis requires further investigation. Enhanced TCA cycle activity not only increases ATP and reducing power for cellular metabolism but also supplies carbon skeletons for the biosynthesis of essential metabolites [68]. Collectively, these results indicate that Se enhances alkaline stress tolerance by maintaining energy metabolism and promoting organic acid accumulation.

Osmoregulation is a key adaptive mechanism under saline–alkali stress. The accumulation of soluble sugars and proline stabilizes proteins and membranes and also contributes to ROS scavenging [30,69]. In this study, NaHCO_3_ stress significantly elevated soluble sugar and proline contents, while Se treatment further enhanced their accumulation, indicating improved osmotic regulation. Sugars not only regulate osmotic pressure but also supply carbon skeletons and energy, while proline provides both osmotic protection and antioxidant activity [69,70]. Similarly, Hawrylak-Nowak (2009) reported that low concentrations of Se increased proline levels, improved growth, and enhanced chlorophyll content in cucumber under NaCl stress [40]. Aquaporins (AQPs), especially plasma membrane intrinsic proteins (PIPs), are also critical for water transport and balance [71]. In this study, NaHCO_3_ stress induced the expression of *PIP1;2* and *PIP2;4* in cucumber roots, and Se further upregulated their expression, suggesting enhanced water transport and efficiency.

Additionally, the role of salicylic acid (SA) is noteworthy. Previous studies show that SA regulates ROS homeostasis and membrane stability [72]. In this study, Se increased both *CsICS* and *CsPAL1* expression and SA levels under alkaline stress. The coordinated regulation of *CsICS* and *CsPAL1,* together with changes in SA accumulation, suggests that Se may help maintain SA biosynthesis under alkaline stress conditions. This indicates that Se’s stress-alleviating effects may partly involve activation of SA biosynthesis. Consistently, Fan et al. (2022) found that Se enhanced drought tolerance in tomato plants by increasing SA content and antioxidant activity [73], and Kowalska et al. (2020) reported that Se interacts with SA to promote sugar accumulation and affects selenium accumulation in lettuce [74].

**Figure 16:**
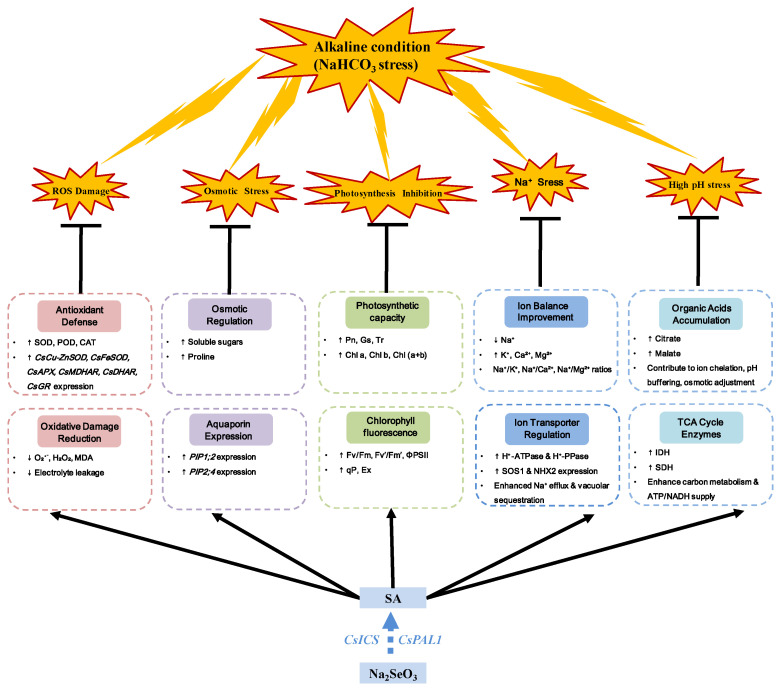
Proposed model illustrating the regulatory role of exogenous Se in enhancing cucumber tolerance to alkaline stress. NaHCO_3_ stress induces multiple adverse effects, including ROS damage, osmotic stress, photosynthetic inhibition, Na^+^ toxicity, and high-pH stress. Exogenous Se counteracts these effects by upregulating *CsPAL1* and *CsICS* expression to promote salicylic acid (SA) accumulation, increasing proline and soluble sugar contents, activating antioxidant defenses, enhancing aquaporin expression and SOS pathway activity, and stimulating organic acid accumulation and TCA cycle enzyme activity. These coordinated regulatory actions alleviate Na^+^ toxicity, oxidative imbalance, and metabolic disruption, ultimately mitigating the growth inhibition of cucumber seedlings under NaHCO_3_ stress.

This study systematically elucidates the mechanisms by which exogenous selenium (Se) alleviates the detrimental effects of NaHCO_3_ stress in cucumber seedlings, as summarized in Figure 16. NaHCO_3_ stress induced multiple adverse effects, including ROS damage, osmotic stress, photosynthetic inhibition, Na^+^ toxicity, and high-pH–related disturbances. Exogenous Se established a multi-tiered defense network by enhancing photosynthetic performance, mitigating oxidative damage, maintaining ion homeostasis, promoting organic acid metabolism and TCA cycle activity, regulating the accumulation of soluble sugars and proline, upregulating aquaporin expression, and activating the salicylic acid (SA) signaling pathway. These coordinated regulatory actions effectively alleviated the physiological and molecular disruptions caused by alkaline stress, thereby significantly improving cucumber growth and tolerance. Overall, the findings provide a comprehensive framework for understanding the protective role of Se under saline–alkali conditions and provide a theoretical basis for developing strategies to improve vegetable production in saline–alkali soils. It should be noted that gene expression was analyzed after 24 h of treatment to characterize the early molecular responses, whereas most physiological and biochemical parameters were determined after 8 days of treatment. Therefore, future studies integrating multiple sampling time points, long-term soil cultivation, and field experiments are required to further validate the practical application of Se under saline–alkali conditions. Such studies will also contribute to the development of integrated physiological and biotechnological strategies for improving crop resilience to saline–alkali stress, as highlighted in recent reviews [75,76].

## 4. Materials and Methods

### 4.1. Plant Material and Experimental Design

Cucumber (*Cucumis sativus* L., cv. ‘Jinyan 4’) seeds with uniform size and vigor were used. Seeds were germinated on moist filter paper in a greenhouse under natural light. At the fully expanded cotyledon stage, seedlings were transferred into 1.0 L plastic pots filled with full-strength Hoagland nutrient solution, with one plant per pot. The nutrient solution was renewed every two days. A total of 30 plants were used for each treatment and randomly arranged. Environmental conditions followed natural light/dark cycles.

The hydroponic system was used in this study to minimize environmental variability and to facilitate precise physiological, biochemical, and molecular analyses during the early response stage of alkaline stress. NaHCO_3_ was selected because it is one of the predominant alkaline salts responsible for saline–alkali stress in agricultural soils and is widely used to simulate alkaline stress conditions in cucumber studies. Sodium selenite (Na_2_SeO_3_) was chosen as the selenium source due to its relatively high bioavailability and frequent application in plant selenium studies. The concentrations of NaHCO_3_ (50 mmol·L^−1^) and Na_2_SeO_3_ (2 μmol·L^−1^) were determined based on preliminary experiments and previous reports, in which the selected NaHCO_3_ concentration induced clear but non-lethal alkaline stress symptoms, while the selected selenium concentration effectively alleviated stress damage without causing selenium toxicity. Four experimental treatments were applied: (1) Control: plants grown in Hoagland solution; (2) Se: Hoagland solution supplemented with 2 µmol·L^−1^ sodium selenite (Na_2_SeO_3_); (3) S: Hoagland solution containing 50 mmol·L^−1^ NaHCO_3_ to impose alkaline stress; (4) S + Se: Hoagland solution with both 50 mmol·L^−1^ NaHCO_3_ and 2 µmol·L^−1^ Na_2_SeO_3_. Treatments were arranged in a completely randomized design with three independent biological replicates. Physiological measurements, including photosynthetic and chlorophyll fluorescence parameters, were performed on randomly selected plants prior to destructive sampling. Independent plants were subsequently harvested for biomass determination and biochemical analyses. For biochemical analyses, tissues from several plants within each biological replicate were pooled to minimize individual variation. The experiment lasted for 8 days for physiological and biochemical analyses, whereas additional short-term sampling (24 h) was performed for gene expression analyses.

Sampling was conducted at multiple time points. Before treatment initiation, samples were collected as the baseline. At 24 h after treatment, root samples were collected for gene expression analyses of ion- and water transport-related genes (*CsSOS1*, *CsNHX2*, *CsPIP1;2*, and *CsPIP2;4*) and antioxidant-related genes (*CsCu/ZnSOD*, *CsFeSOD*, *CsAPX*, *CsMDHAR*, *CsDHAR*, and *CsGR*), whereas leaf samples were collected for the analysis of salicylic acid-related genes (*CsICS* and *CsPAL1*). The 24 h sampling point was selected to evaluate early molecular responses to alkaline stress and selenium treatment. Final physiological and biochemical measurements were performed after 8 days of treatment, including phenotype photography, shoot and root dry weights, root activity, photosynthetic and chlorophyll fluorescence parameters, ROS accumulation, MDA content, electrolyte leakage, antioxidant enzyme activities, osmolyte accumulation, ion contents, proton pump activities, TCA cycle enzyme activities and organic acid contents.

### 4.2. Growth and Root Activity

Growth measurements: At harvest, shoots and roots were separated and dried in an oven at 105 °C for 10 min, then at 75 °C until constant weight. Shoot and root dry weights were recorded to calculate total biomass.

Root activity, which reflects the overall metabolic and respiratory capacity of root tissues, was determined using the triphenyltetrazolium chloride (TTC) reduction method. Fresh root segments (approximately 0.5 g) were incubated in TTC solution at 37 °C for 2 h in darkness. Living root tissues reduce TTC to red-colored triphenylformazan (TTF) through dehydrogenase activity. The produced TTF was extracted with ethyl acetate, and absorbance was measured at 485 nm. Root activity was expressed as mg TTC reduced g^−1^ FW h^−1^.

### 4.3. Chlorophyll Content

Fresh leaf samples (approximately 0.1 g) were homogenized and extracted with acetone according to the method of Lichtenthaler (1987) [77]. After centrifugation, the absorbance of the supernatant was measured spectrophotometrically at specific wavelengths, and the contents of chlorophyll a (Chl a), chlorophyll b (Chl b), and total chlorophyll (Chl a + b) were calculated using Lichtenthaler’s equations. Chlorophyll content was expressed on a fresh weight basis.

### 4.4. Chlorophyll Fluorescence

Chlorophyll fluorescence parameters were measured using a PAM-2500 pulse-amplitude modulated fluorometer (Heinz Walz GmbH, Effeltrich, Germany). Prior to measurement, leaves were dark-adapted for 20 min to ensure full oxidation of PSII reaction centers. The maximum photochemical efficiency of PSII (Fv/Fm), actual photochemical efficiency of PSII (ΦPSII), efficiency of excitation energy capture by open PSII reaction centers (Fv′/Fm′), photochemical quenching coefficient (qP), and excitation energy capture efficiency (Ex) were recorded according to the manufacturer’s instructions.

### 4.5. Determination of ROS, Electrolyte Leakage and MDA

The production rate of superoxide anion (O_2_·^−^) and the content of hydrogen peroxide (H_2_O_2_) were determined according to the methods of Gong et al. (2014) [66]. For histochemical staining, leaf disks of identical diameter were excised from comparable positions of fully expanded leaves while avoiding the main veins to minimize tissue heterogeneity. All leaf disks were stained and photographed under identical experimental conditions. Malondialdehyde (MDA) content was measured as an indicator of membrane lipid peroxidation, and electrolyte leakage was determined to evaluate membrane permeability using previously described methods [51,66].

### 4.6. Antioxidant Enzyme Assays

Fresh cucumber leaf and root tissues (approximately 0.1 g) were homogenized in pre-chilled phosphate buffer under ice-cold conditions and centrifuged to obtain enzyme extracts. The resulting supernatants were used for the determination of superoxide dismutase (SOD), peroxidase (POD), and catalase (CAT) activities according to previously described methods [51,78]. Soluble protein concentration was quantified for normalization, and enzyme activities were expressed on a protein basis (U mg^−1^ protein).

### 4.7. Determination of Mineral Element Content

Dried leaf and root samples were ground into a fine powder and digested according to previously described methods [79]. The concentrations of Na^+^, K^+^, Ca^2+^, and Mg^2+^ were subsequently determined using an atomic absorption spectrometer (ZEEnit 700P, Analytik Jena AG, Jena, Germany).

### 4.8. Determination of TCA Cycle Enzyme Activity and Organic Acid Content

The activities of isocitrate dehydrogenase (IDH) and succinate dehydrogenase (SDH) were determined according to the method of Zhong et al. (2016) [80].

For the determination of organic acids, the contents of citric acid and malic acid were analyzed following the method of Guo et al. (2018) [81].

### 4.9. Gene Expression Analysis

Total RNA was isolated from cucumber samples, and first-strand cDNA was synthesized using commercial kits (Vazyme Biotech Co., Ltd., Nanjing, China). Quantitative real-time PCR (qRT-PCR) was performed using an ABI 7500 Real-Time PCR System (Applied Biosystems, Foster City, CA, USA) with gene-specific primers listed in Table 2. 

### 4.10. Statistical Analysis

All experiments were conducted using three independent biological replicates. Data are presented as the mean ± standard deviation (SD). Statistical analyses were performed using IBM SPSS Statistics 26.0 (IBM Corp., Armonk, NY, USA). Differences among treatments were analyzed by one-way analysis of variance (ANOVA) followed by Duncan’s multiple range test. Differences were considered statistically significant at *p* < 0.05.

## Figures and Tables

**Table 1:** Effects of Se on chlorophyll fluorescence indices in cucumber leaves under NaHCO_3_ stress.

Treatments	Fv/Fm	Fv′/Fm′	ΦPSII	qP	Ex
Control	0.83 a	0.80 a	0.99 a	0.99 a	4.26 a
Se	0.82 a	0.81 a	0.98 a	0.97 a	4.11 a
S	0.31 c	0.19 c	0.63 c	0.37 c	2.61 b
S + Se	0.57 b	0.51 b	0.89 b	0.48 b	3.39 ab

Notes: Fv/Fm = maximum quantum efficiency of PSII photochemistry; Fv′/Fm′ = efficiency of excitation energy capture by open PSII reaction centers; ΦPSII = effective quantum yield of PSII photochemistry; qP = photochemical quenching coefficient; Ex = excitation energy flux. Treatments: Control, Hoagland solution; Se, Hoagland + Na_2_SeO_3_; S, Hoagland + NaHCO_3_; S + Se, Hoagland + NaHCO_3_ + Na_2_SeO_3_. Values are presented as the mean ± SD (*n* = 3). Different lowercase letters indicate significant differences among treatments at *p* < 0.05 according to Duncan’s multiple range test.

**Table 2:** Primer sequences used in this study.

Gene	Forward Primer (5′ → 3′)	Reverse Primer (5′ → 3′)
*Actin*	CCCCGATGGGCAGGTAATA	AAGAGCAGGACGAACAGCAGA
*SOS1*	ATCCAACGGAGTGTGTTAAA	AACAACGGAATCTGTAATC
*NHX2*	AGGGTGTAGTGAATGACG	GAGAATGCCACTCAAATC
*PIP1;2*	CATTATTTACAACCCAGCAGAAGCA	GGATTGAAGAAGCATCATGGATTAGA
*PIP2;4*	GCTGCTCTGCTCTCATCTTGC	GAAAATACATGAATAACAGGAGCCCC
*CsICS*	GTTCCAATCCAACAACGAAT	CTTGATAGACGCCCAATCG
*CsPAL1*	ATGGCTTCATATTGCTCTGAG	ATGCCTCAAGTCAATTGCTTG
*CsCu*/*ZnSOD*	CTCCATTTTCAATCTCATTCATTC	ATAGAAGTGATTGTGCGCCATAG
*CsFeSOD*	TACACTGAACCTGAGTTCAAACAC	ACGTTCCTCGGAAACCAAACAGA
*CsAPX*	CTGCTACTGTTTGGACCAACGC	GCGGAGGAGGAAACGGTAGTT
*CsMDHAR*	ACAGCCTTCTTCTGTTGCCTCAG	CTCTATTGTCGTTCGGAATACCGC
*CsDHAR*	ATGTCGGGCTCCAGAATCCACCA	AAAGCGAAGTGAAGGAAGGAGT
*CsGR*	GTCCGGAATGGCTGGAGGTTGG	CCATCCAAAGCCATGACTCTTC

## Data Availability

The original contributions presented in this study are included in the article. Further inquiries can be directed to the corresponding authors.
